# The native T_1_ in remote myocardium of patients with prior chronic infarction is not normal

**DOI:** 10.1186/1532-429X-18-S1-P102

**Published:** 2016-01-27

**Authors:** Steven Bellm, Shingo Kato, Ravi V Shah, Sophie Berg, Kraig V Kissinger, Beth Goddu, Long Ngo, Warren J Manning, Reza Nezafat

**Affiliations:** grid.239395.70000000090118547Medicine, Beth Israel Deaconess Medical Center, Boston, MA USA

## Background

Global left ventricular (LV) remodeling after myocardial infarction frequently occurs. Late Gadolinium Enhancement (LGE) CMR allows imaging of focal myocardial scar with areas remote from scar having no hyperenhancement. Myocardial T_1_ mapping allows quantification of interstitial fibrosis and may be a surrogate for LV remodeling. We sought to determine if there were T_1_ abnormalities in remote regions (no LGE positive areas) in patients with prior myocardial infarction.

## Methods

In a prospective IRB-approved study, 12 patients with a history of coronary artery disease (CAD) and chronic myocardial infarction (61 ± 9 years, 9 males) and 10 healthy subjects (52 ± 10 years, 8 males) were recruited to undergo CMR scans. All subjects were in sinus rhythm during CMR study. We assessed native T_1_ mapping using the slice interleaved T_1_ sequence in 5 short axis-slices (from apical to basal). The sequence was acquired in a free-breathing ECG-triggered slice-selective bSSFP. T_1_ mapping of each scan was estimated by voxel-wise curve fitting using a 2-paramter fit model. All images were corrected for in-plane motion between different T_1_ weighted scans. Native myocardial T_1_ in healthy subjects were measured over the three mid-ventricular slices by manually drawing epicardial and endocardial contours. The native T_1_ times of the remote myocardium of the CAD patients were measured by manually drawing a region of interest (ROI) on the three mid-ventricular slices and excluding the infarct area. An unpaired-samples T-test analysis was used to test for statistically significant differences between the two groups.

## Results

**Figure 1:**
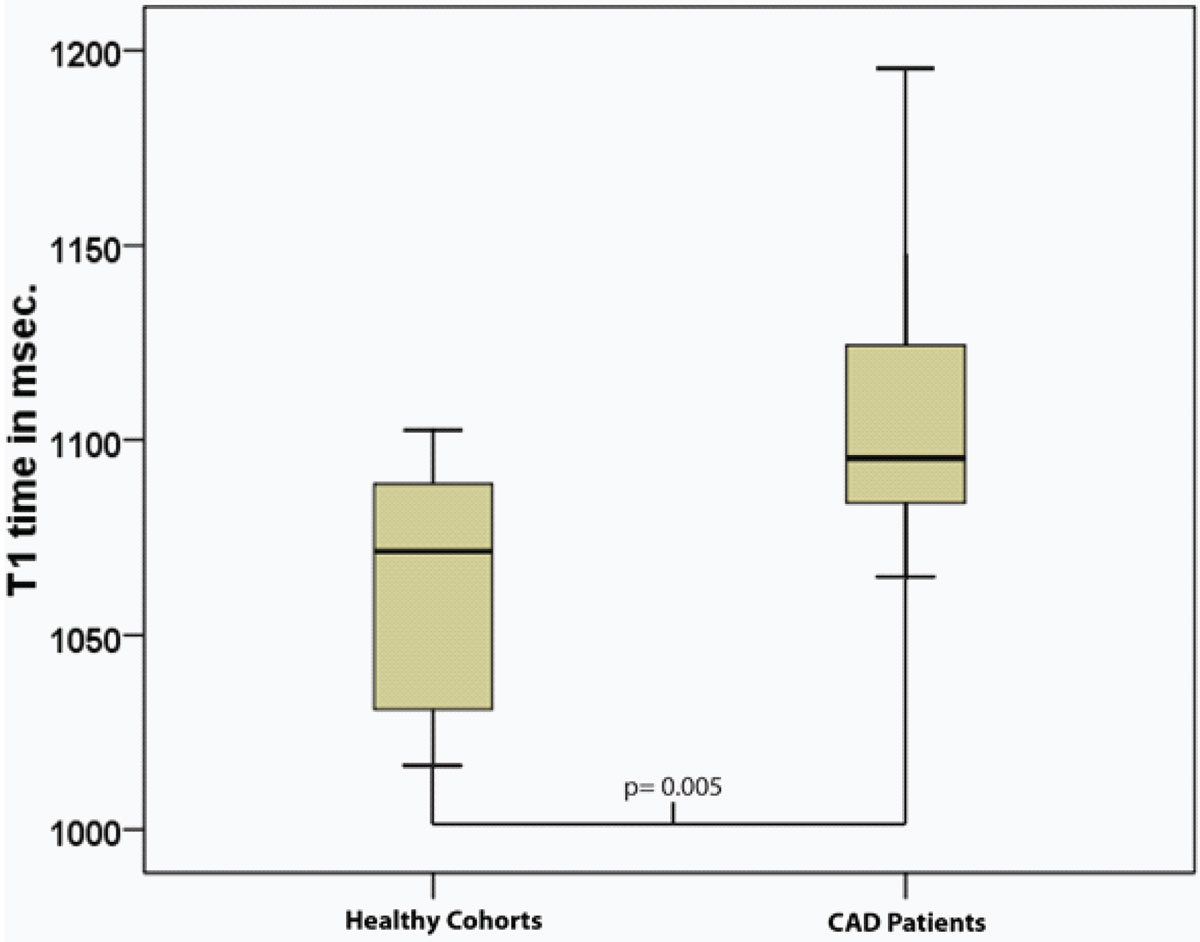
**boxplot with mean (line in box), interquartile range (box) and complete range (whisker) of native T1 time in remote myocardium of CAD patients and healthy cohorts (p = 0.0005)**.

Patient characteristics are summarized in Table 1. LGE hyperenhancement was observed in all CAD patients. The mean native T_1_ time in the remote area myocardium of the CAD patients was significantly *higher* than the native T_1_ value of the myocardium in the healthy group (1107 ± 36 ms. vs. 1061 ± 32 ms.; p = 0.005) (Figure 1).

**Table 1:** Subject characteristics of CAD patients and healthy cohorts with p-values of comparison.

	CAD Patients (N = 12)	Healthy Cohorts (N = 10)	Comparison (p-value)
Male, %	75% (N = 9)	80% (N = 8)	
Age, years	61 ± 9	52 ± 10	0.029
Height, cm	174 ± 8	174 ± 8	0.936
Weight, kg	82 ± 18	81 ± 17	0.893
Hypertension, %	75% (N = 9)		
Type 2 Diabetes, %	25% (N = 4)		
BSA, m2	1.99 ± 0.24	1.98 ± 0.25	0.918
SBP, mmHg	115 ± 16	112 ± 14	0.63
DBP, mmHg	64 ± 12	61 ± 11	0.633
HR, beats per minute	64 ± 11	58 ± 9	0.188
EDV-Cine, ml	231 ± 61	175 ± 27	0.011
ESV-Cine, ml	146 ± 52	73 ± 19	0.0001
SV-Cine, ml	84 ± 24	102 ± 9.5	0.033
EF-Cine, %	37 ± 9	59 ± 5	0.0001
LV-mass, grams	128 ± 32	102 ± 14	0.022

## Conclusions

Our data suggest there are diffuse changes in remote/normal myocardium resulting in abnormal/higher native T_1_ times in CAD patients with prior myocardial infarction. Further studies are needed to assess the prognostic value of an abnormal native T_1_ in the remote region among CAD patients.

